# Perceived urban environment elements associated with momentary and long-term well-being: An experience sampling method approach

**DOI:** 10.1038/s41598-025-88349-x

**Published:** 2025-02-05

**Authors:** Yu-Ru Chen, Atsushi Nakagomi, Masamichi Hanazato, Noriyuki Abe, Kazushige Ide, Katsunori Kondo

**Affiliations:** 1 Department of Social Preventive Medical Sciences, Center for Preventive Medical Sciences, Chiba University, 1-33 Yayoi-cyo, Inage-ku, Chiba-shi, Chiba, 263-8522 Japan; 2 Department of Building Community for Well-being, Center for Preventive Medical Sciences, Chiba University, Chiba, Japan; 3 Social Insurance and Welfare, Institute for Health Economics and Policy, Tokyo, Japan

**Keywords:** Positive psychology, Urban design, Structural equation model, Exploratory factor analysis, Epidemiology, Environmental impact, Psychology and behaviour

## Abstract

Well-being (WB) extends beyond physical health, as defined by the World Health Organization and encompasses two aspects: (1) long-term WB (LWB), such as overall life satisfaction; and (2) momentary WB (MWB), such as immediate mood. While research has demonstrated a positive association between one’s environment and LWB, limited studies have explored the association between environmental characteristics and MWB. We applied the experience sampling method (ESM) to collect data on location, perceived environmental characteristics, MWB, and LWB of adult participants living in Kashiwa-no-ha area, Kashiwa City, Japan. Structural equation modeling was used to calculate the correlation coefficients between different environments and both MWB and LWB. LWB was positively associated with MWB (standardized coefficient=0.24) and urban elements—cafés/restaurants/bars (0.11), cultural/sports/education facilities (0.04), and public spaces (0.11). Meanwhile, relaxing/clean (0.56) and natural environments (0.14) were associated with higher MWB. Conversely, vibrant environments (−0.14) and being on the move (−0.11) were linked to lower MWB. LWB was positively associated with being at cultural/sports/educational facilities (0.06), and vibrant (0.15) and communicative (0.13) urban settings. MWB was associated with various built environments, emphasizing the need for places like parks and cafés that promote a positive mood, and well-designed transportation and roads. These elements are important for developing urban areas that meet the physical requirements of residents while supporting their emotional and psychological well-being.

**Supplementary Information:**

The online version contains supplementary material available at 10.1038/s41598-025-88349-x.

## Introduction

Positive psychology represents a shift from traditional health models that focus on the absence of illness; instead, it focuses on a positive understanding of health, including emotional and psychological thriving^1^. One of the key aspects of positive psychology is subjective well-being, which refers to an individual’s perception of their own well-being encompassing various elements such as life satisfaction, happiness, purpose of life, and meaning in life^1–3^. These elements are intrinsically linked to personal health and vitality^1–4^.

Understanding the various aspects of subjective well-being from a temporal perspective is essential, highlighting the importance of both long-term well-being (LWB) and momentary well-being (MWB). LWB reflects overall cognition of life satisfaction and mental health over extended periods, while MWB, a multidimensional concept similar to mood, captures the relatively immediate emotional responses to daily experiences^5–7^. According to emotional theories, these moment-to-moment experiences of emotions are interconnected with fulfillment, engagement, and self-concept^8^. While the enduring nature of LWB provides a stable backdrop for life satisfaction, the variable experiences of MWB contribute dynamically to our ongoing assessments and experiences of well-being^8^. Previous studies have identified a relationship between LWB and personality traits^9^. Economic and social resources, such as social capital, cultural values, and various dimensions, were identified as crucial predictors of changes in LWB over time^10^. Regarding MWB, factors such as comfort and security are significantly related to situational variables and environmental characteristics, including the type of activity, place characteristics, and the company one keeps^5^. Few studies investigated the relationship between LWB and MWB, with MWB assessed through multiple scales on respondents’ feelings, such as security and comfort during momentary experiences. Their findings revealed that LWB is positively associated with MWB; this suggests that individuals with higher levels of LWB are more likely to experience higher levels of MWB, indicating a directional relationship from LWB to MWB^11^.

Urban environment elements play a significant role in LWB; however, research on MWB in this context is limited, and existing studies often face methodological limitations. A systematic review provided strong evidence that specific natural features like trees or vegetation cover reduce stress, increase satisfaction, and improve mental well-being^12^. Higher health-related quality of life is associated with lower annoyance from transportation noise, greater satisfaction with the living environment, and improved access to green spaces^13^. Other urban environmental features, such as land use mix and proximity to amenities, are also significantly correlated with life satisfaction^7^. On the other hand, studies from Israel, Europe, and China have examined the link between the urban environment and MWB, revealing the significance of environmental factors, such as nature/open spaces, convenient/leisure facilities, or accessibility of shops^5,7,11,14–16^. Additionally, better accessibility to a location reduces feelings of loneliness^16^. However, these studies regarding MWB have limitations, such as focusing only on student or specific populations^5,14,16^ or using single-item scales to measure MWB, thereby overlooking its multidimensional nature^7^. Additionally, there is a scarcity of research investigating detailed characteristics, such as the atmosphere and perception of locations, natural environmental objects, and artificial physical objects, or urban planning features within the study area^11^. While objective environmental characteristics are essential, environmental psychology highlights that the perceived environment also plays a critical role in shaping individuals’ interactions, evaluations, and behaviors within their surroundings^17^. For instance, nature has been reported to enhance feelings of tranquility and pleasure through soundscapes, contributing to restorative experiences^18^. As the study described, the perceived environment triggers sensory perceptions, such as visual and auditory stimuli, which interact with cognitive and emotional processes to promote psychological stability and well-being.

Therefore, this study aims to analyze the relationship between perceived urban environmental elements and both MWB and LWB among adults. In this study, we examine the relationship between urban environments and well-being while considering individual characteristics, this study also integrates personal attributes into its examination to uncover how these relationships are associated with individual characteristics and contextual factors within the urban setting^11,15^. Our study focuses on the following three research questions:

1. What is the relationship between MWB and LWB?
2. What are the urban environmental elements and personal characteristics associated with MWB?
3. What are the urban environmental elements and personal characteristics associated with LWB?

## Methods

### Datasets and study participants

We targeted people aged 18 or older who lived in or visited the Kashiwa-no-ha Campus Station area (Kashiwa-no-ha area) in Kashiwa City, Chiba Prefecture. Located in the suburban Tokyo metropolitan area, the Kashiwa-no-ha district is home to 13,369 residents as of 2023 and was developed in 2005 ^19^. It is known for its innovative approach to urban living, particularly in the fields of education and research^20^. For participant recruitment, flyers were distributed to residences around the station, information was shared through available channels such as event announcements in Kashiwa-no-ha and local information apps, and cooperation for promotion was obtained from elementary schools and stores. Two types of online surveys (A. Online questionnaire, B. Experience Sampling Method survey) were conducted using a research account on the smartphone application LINE. These two types of surveys are further detailed below.

A. Online questionnaire (conducted from April 4 to 30, 2022): Survey items included individual characteristics, personal behavior, LWB, psychological factors, and physical factors.
B. Experience Sampling Method survey (conducted from May 14 to June 5, 2022): Participants in the online questionnaire were targeted using the Experience Sampling Method (ESM), which is an intensive and longitudinal approach that can be referred to as the diary method or ecological momentary assessment. ESM, unlike single-time point measurements, reduces recall bias by asking participants to respond to their thoughts, feelings, behaviors, and environment in a variety of events/situations/moments (hereafter “events,” such as shopping or exercising, and so on) over time^21,22^. We asked the participants to report, using the LINE app, with the following details whenever they were experiencing various events in urban environments: MWB, the place where the event occurred and the characteristics of the place, whether the experience was indoor or outdoor, the time of day when the event occurred, and so on. There was no cap on the number of reports that respondents could send. To stimulate report-sending, the participants received four weekly reminders from May 14 to June 5, 2022. To encourage participation in both the online questionnaire and ESM surveys, respondents were offered a 1500 yen gift voucher.

**Fig. 1:**
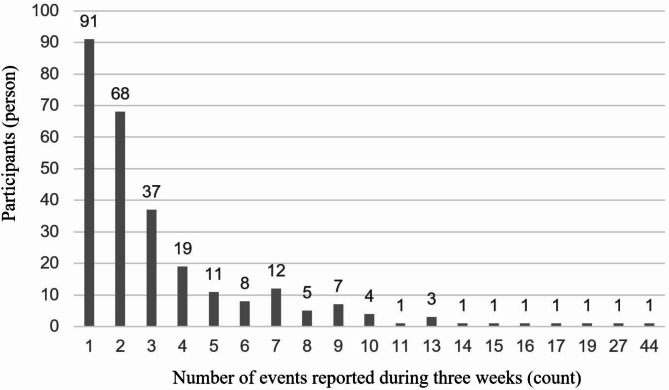
Distribution of the number of events reported.

The online questionnaire received 433 valid responses, and the ESM survey had 306 valid participants, resulting in a total of 988 reports. After excluding responses with missing household income data, the final number of participants was 273, accounting for 900 reports. On average, each participant reported 3.3 events over the three weeks, with a minimum of 1 and a maximum of 44 events, with a standard deviation of 3.9. The distribution of the number of reported events is shown in Fig. 1. This study was approved by the Ethical Review Committee of Chiba University (Approval No.: M10253). All methods were conducted according to relevant guidelines and regulations or according to the Declaration of Helsinki. All participants gave their online informed consent before participating in the study.

### Variables measured

The variables used were: (1) LWB, (2) MWB, (3) The place where the event occurred (hereafter “types of locations”), (4) The characteristics of the place where the event occurred (hereafter “environmental characteristics”), and (5) Individual characteristics and health status (variables 1 and 5 were obtained from the online questionnaire, and variables 2–4 were obtained from the ESM survey). The definitions of the variables are as follows:

#### Long-term well-being (LWB)

Four variables— Life satisfaction, happiness, purpose in life, and meaning of life—were rated on a scale from 0 to 10, where 10 represented the most ideally positive state and 0 the least favorable or worst state.

#### Momentary well-being (MWB)

To evaluate MWB, a brief six-item scale from the Multidimensional Mood Questionnaire was used, particularly adapted for use in smartphone-based assessments of MWB in ecological settings^21,23^. To minimize the burden on participants, we chose a scale with a limited number of questions that is widely used and capable of measuring both positive and negative aspects of mood. This scale measures three affective states: valence, calmness, and energetic arousal, with two bipolar scales for each dimension. The valence scale measures well/unwell and contentment/discontentment. The calmness scale ranges from relaxed to tense, and calm to agitated. Energetic arousal is measured from tired to awake, and from without energy to full of energy. Each bipolar scale has seven points, ranging from 0 to 6, with 0 being the least and 6 representing the most for the respective feeling.

#### Types of locations

Location of events was classified into seven categories: (1) Workplace, (2) On the move, (3) Shopping mall/store, (4) Cafe/restaurant/bar, (5) Cultural/sports/educational facility, (6) Public space (such as a park or plaza), and (7) Other. Regarding the selection of types of locations, the goal was to provide insights that enable stakeholders in urban design, such as the private sector, public sector, and academic institutions, to make informed decisions prioritizing developments that contribute to well-being. Therefore, we included all spaces outside the home, such as public spaces and semi-public or semi-private spaces, where individuals spend a significant portion of their daily time^5,11^.

####  Perceived environmental characteristics

Perceived environmental characteristics were subjectively evaluated using 15 descriptors: (1) lively and crowded, (2) meeting familiar people, (3) pleasant design and atmosphere of the space, (4) easy to have a conversation, (5) perceived safety, (6) clean, (7) greenery such as trees and plants, (8) waterfront such as a river or the ocean, (9) pleasant natural light (e.g. sunlight filtering through trees), (10) pleasant artificial light (e.g., color and arrangement of lighting), (11) benches and chairs to sit or rest, (12) signs providing directions/information about the location, (13) pleasant sounds and music, (14) parking lots and bicycle parking areas, and (15) easy access. These descriptors were ranked from 0 to 3, where 0 meant strongly disagree and 3 meant strongly agree. The selected items encompass environmental characteristics that address both indoor and outdoor spaces, focusing primarily on visual elements, as Gehl^24^ indicated that vision plays a dominant role in human perception of urban spaces. In addition, auditory elements were chosen based on insights from previous studies, while other elements were selected based on the urban design guidelines of the study area, specifically the Kashiwa-no-ha Campus^5,11,24,25^.

#### Individual characteristics and health status

Individual characteristics and health status were gender (male, female, prefer not to answer), age, household income (< JPY 2, 2–3.99, 4–5.99, 6–7.99, 8–9.99, 10–14.99 or ≥ 15 million per year), years of residence, psychological stress, and self-rated health (healthy or unhealthy). Psychological stress was evaluated by the Kessler 6 scale (K6)^26^.

### Statistical analysis

Descriptive analysis, exploratory factor analysis, and Structural Equation Model (SEM) were used for data analysis. Following previous research, we combined indoor and outdoor experiences^11^. For the 15 items of perceived environmental characteristics, exploratory factor analysis was conducted, and the resulting synthetic variables were tested for reliability. The number of factors was determined based on the Guttman and Scree criteria, and the final factors were determined by excluding items with factor loadings of 0.4 or less. SEM represents an advanced form of multiple regression analysis and structural equation modeling, that includes observed or measured variables^27^. As observed variables, we generated LWB (life satisfaction, happiness, purpose in life, and meaning of life) and MWB (valence, calmness, and energetic arousal). The measured variables were types of locations, factors of perceived environmental characteristics, individual characteristics, and health status, comprising only continuous and dummy variables. The categorical variables (e.g., types of locations) were dummy-coded into binary variables to allow their inclusion in the SEM. To construct the model’s framework, we employed a stepwise model selection technique along with the robust maximum likelihood method. This approach involved incorporating observed and measured variables, as well as their relationships, which were deemed potentially significant based on literature review findings and were substantiated through bivariate analyses. The model permits correlations between variables. In order to reduce the number of variables in the SEM and not fitting the model, the links that were not found to be significant in the SEM with respect to the 0.05 significance level were subsequently removed. This process was repeated until a model was estimated with only significant relationships and the best model fit. For our analyses, we utilized R version 4.3.1 and Mplus version 8.10.

## Results

### Descriptive analysis

The main characteristics of the 273 participants are as follows: the majority were women (59%), with the largest age group being 36–45 years old (29.7%). Most earn between 10 and 14.99 million yen per year (28.6%) and have resided in their current location for 0–3 years (32.2%). Additionally, a high percentage did not experience high levels of psychological distress (77.7%). Regarding LWB, as shown in Table 1, “happiness” emerges as the highest value, followed by meaning of life, life satisfaction, and purpose in life. In terms of MWB, detailed in Table 1, the average score and standard deviation scores for valence, calmness, and energetic arousal are 9.15 ± 3.02, 9.26 ± 2.43, and 8.57 ± 2.5, respectively.

Regarding characteristics of the 900 events (Table 2), of which 59.4% involved outdoor experiences, most observations occurred in public spaces (32.2%), followed by shopping malls/stores, cafes/restaurants/bars, on the move, cultural/sports/educational facilities, workplaces, and others. For environmental factors, the means that were above 2.0 include pleasant design and atmosphere of the space (2.14 ± 0.82), perceived safety (2.31 ± 0.73), clean (2.21 ± 0.72), greenery such as trees and plants (2.31 ± 0.9), pleasant natural light (2.05 ± 0.97), and easy to access (2.49 ± 0.7).

**Table 1:** Characteristics of the study participants (*n* = 273).

	Total	
	n	%
Gender		
Men	111	40.7
Women	161	59.0
Prefer not to answer	1	0.4
Age group, years		
18–35	49	17.9
36–45	81	29.7
46–55	57	20.9
56–64	32	11.7
> 65	54	19.8
Household income, million yen		
< 2.00	13	4.8
2.00–3.99	20	7.3
4.00–5.99	43	15.8
6.00–7.99	43	15.8
8.00–9.99	47	17.2
10–14.99	78	28.6
≥ 15	29	10.6
Years of residence		
0–3	88	32.2
4–6	58	21.2
7–10	41	15.0
11–13	38	13.9
14–16	25	9.2
> 16	23	8.4
Subjective health status		
Healthy	250	91.6
Unhealthy	23	8.4
High levels of psychological distress (K6 ≥ 13)		
No	212	77.7
Yes	61	22.3
Long-term well-being	Mean	St. deviation
Life satisfaction	7.36	1.82
Happiness	7.87	1.69
Purpose in life	6.67	2.38
Meaning of life	7.69	1.89
Momentary well-being	Mean	St. deviation
Valence	9.15	3.02
Unwell/well	4.62	1.52
Discontentment/contentment	4.53	1.57
Calmness	9.26	2.43
Relaxed/tense	4.67	1.28
Calm/agitated	4.59	1.27
Energetic arousal	8.57	2.5
Tired/awake	4.31	1.32
Without energy/full of energy	4.25	1.26

**Table 2:** Characteristics of the events (*n* = 900).

	Total	
	n	%
Indoor/Outdoor		
Indoor	365	40.6
Outdoor	535	59.4
Location type		
Workplace	9	1.0
On the move	121	13.4
Shopping mall/store	171	19.0
Cafe/restaurant/bar	123	13.7
Cultural/sports/educational facility	61	6.8
Public space	290	32.2
Other	125	13.9
Perceived environmental characteristics	Mean	St. deviation
Lively and crowded	1.73	0.94
Meeting familiar people	1.18	0.95
Pleasant design and atmosphere of the space	2.14	0.82
Easy to have a conversation	1.94	0.88
Perceived safety	2.31	0.73
Clean	2.21	0.72
Greenery such as trees and plants	2.31	0.9
Waterfront such as a river or the ocean	1	1.19
Pleasant natural light (e.g. sunlight filtering through trees)	2.05	0.97
Pleasant artificial light (e.g. color and arrangement of lighting)	1.59	0.98
Benches and chairs to sit or rest	1.92	0.94
Signs providing directions/information about the location	1.72	0.89
Pleasant sounds and music	1.26	0.95
Parking lots and bicycle parking areas	1.8	0.97
Easy to access	2.49	0.7

### Exploratory factor analysis

After conducting exploratory factor analysis twice, four factors were extracted. In the first factor analysis, “easy to access” was excluded due to its factor loadings being below 0.4. The results of the second factor analysis, highlighted in pink in Table 3, indicate that Factor 1 (F1) consisted of 6 items: lively and crowded, pleasant artificial light, benches and chairs to sit or rest, signs providing directions/information about the location, pleasant sounds and music, and parking lots and bicycle parking areas; this factor was labeled “Vibrancy.” Factor 2 (F2) had 3 items for greenery such as trees and plants, waterfront such as a river or the ocean, and pleasant natural light, leading to its label “Nature.” Factor 3 (F3) comprised 3 items: pleasant design and atmosphere of the space, perceived safety, and clean, and was labeled “Relaxing and clean.” Lastly, Factor 4 (F4) consisted of 2 items: meeting familiar people and easy to have a conversation and was labeled “Communication.”

**Table 3:** Results of the second factor analysis.

Perceived environmental characteristics	F1: Vibrancy	F2: Nature	F3: Relaxing and clean	F4: Communication
Lively and crowded	0.448	−0.178	−0.288	0.242
Pleasant artificial light (e.g. color and arrangement of lighting)	0.6	−0.121	0.167	−0.027
Benches and chairs to sit or rest	0.552	0.213	0.069	0.007
Signs providing directions/information about the location	0.739	0.114	−0.037	−0.053
Pleasant sounds and music	0.524	0.038	0.051	0.069
Parking lots and bicycle parking areas	0.465	−0.028	0.048	0
Greenery such as trees and plants	−0.094	0.852	−0.007	0.026
Waterfront such as a river or the ocean	0.183	0.503	−0.115	−0.059
Pleasant natural light (e.g. sunlight filtering through trees)	−0.039	0.921	−0.018	−0.008
Pleasant design and atmosphere of the space	0.242	0.133	0.541	0.016
Perceived safety	−0.08	0.055	0.709	0.166
Clean	0.043	−0.156	0.968	−0.111
Meeting familiar people	0.092	−0.054	−0.081	0.524
Easy to have a conversation	−0.037	0.044	0.152	0.722

*F* Factor

Factor extraction method: Maximum likelihood method

Rotation method: Promax method with Kaiser normalization

### SEM

**Fig. 2:**
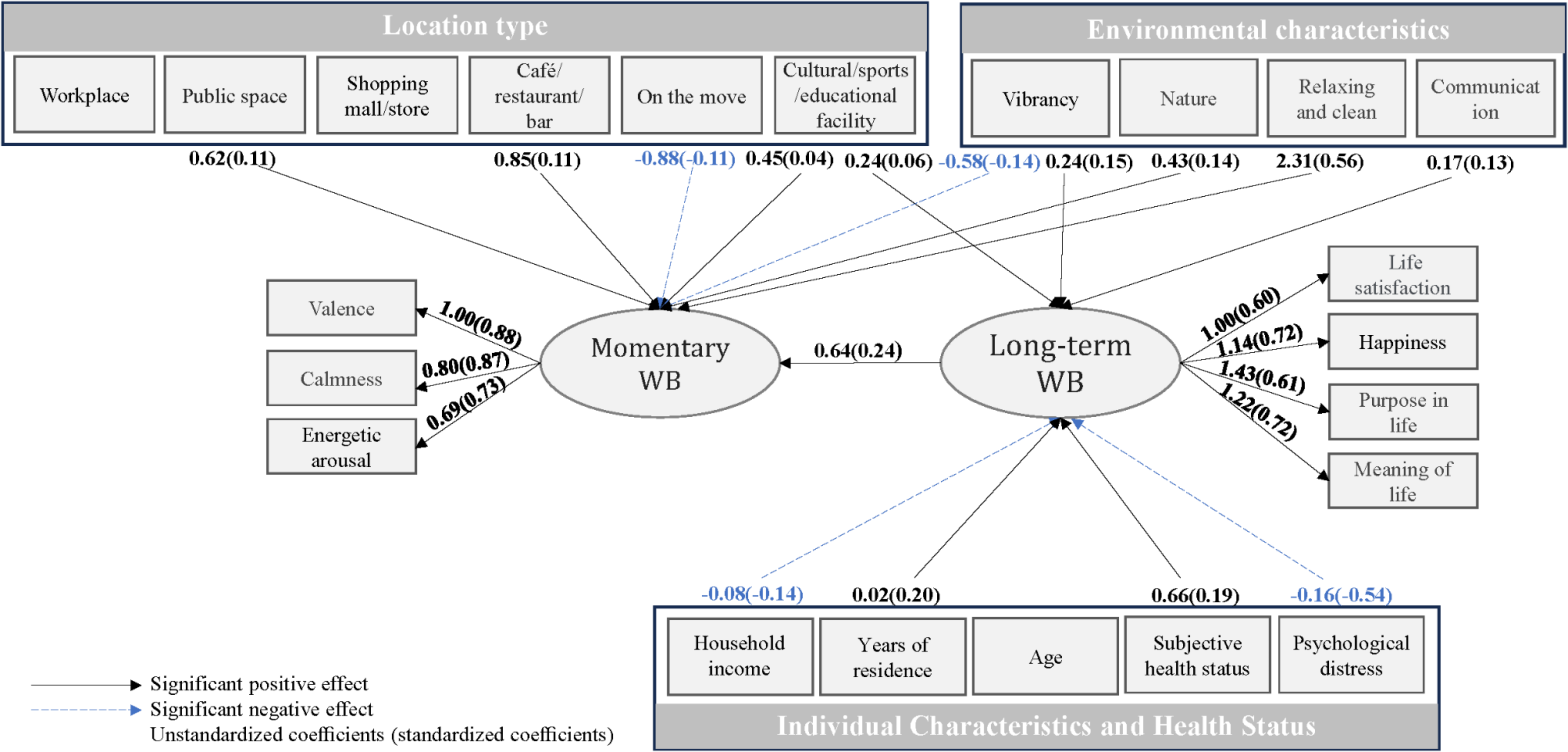
Significant results of SEM (*n* = 900).

The model’s fit with the data is presented in Table 4. The model demonstrates an adequate fit, as indicated by the following indices: the root mean square error of approximation (RMSEA) is 0.065, suggesting a reasonable error level; the Comparative Fit Index (CFI) stands at 0.864; the Tucker-Lewis Index (TLI) is 0.875; and the Standardized Root Mean Square Residual (SRMR) is 0.057, further supporting the appropriateness of the model. While the ratio of the Chi-Square to degrees of freedom does not approach 1, the significance level is below 0.001, indicating an acceptable model fit. Table 5; Fig. 2 show the results of the path model (unstandardized and standardized estimates).

In addressing our three research questions, the results (unstandardized coefficients and standardized estimates) from the path analysis can be interpreted as follows:

#### What is the relationship between MWB and LWB?

LWB positively affects MWB, with an unstandardized coefficient of 0.64 and a standardized coefficient of 0.24, respectively. This suggests that higher levels of LWB are associated with increased MWB.

#### What are the urban environmental elements and individual characteristics associated with MWB?

In terms of types of locations, cafe/restaurant/bar (coefficients = 0.85, standardized coefficients = 0.11), culture/sport/education facility (0.45, 0.04), and public space (0.62, 0.11) exhibit positive relationships with MWB. Additionally, environments perceived as nature (0.43, 0.14) and as being relaxing and clean (2.31, 0.56) are positively associated with MWB. Conversely, being on the move (-0.88, -0.11) and being in environments with vibrancy (−0.58, −0.14) are negatively associated with MWB. Individual characteristics do not appear to be directly associated with MWB within the scope of this model.

####  What are the urban environmental elements and individual characteristics associated with LWB?

Culture/sport/education facility (0.24, 0.06) has a positive relationship with LWB; also, vibrancy (0.24, 0.15) and communication (0.17, 0.13) in the urban environment are positively related. Regarding individual characteristics, years of residence (0.02, 0.20) and self-rated health (0.66, 0.19) have positive associations with LWB, while household income (−0.08, −0.14) and high psychological distress (−0.16, −0.54) show negative relationships with LWB.

**Table 4:** Model fit information.

Root mean square error of approximation (RMSEA)	0.065
90% confidence interval for RMSEA	0.060; 0.069
p-value for test of close fit (RMSEA < 0.05)	< 0.001
Comparative fit index (CFI)	0.864
Tucker-Lewis index (TLI)	0.875
Standardized root mean square residual (SRMR)	0.057
Chi-square value	729.8
Degrees of freedom	153
p-value	< 0.001

The RMSEA is required to be less than 0.1, and a value of less than 0.05 is considered to be a fairly good model fit. For CFI/TLI, a value of 0.9 or higher is considered good and 0.95 or higher is considered fairly good. For SRMR, a value of less than 0.05 is considered to be a fairly good model fit.

**Table 5:** SEM results (*n* = 900).

	Momentary WB		Long-term WB	
	Coefficients (standardized coefficients)	p-value	Coefficients (standardized coefficients)	p-value
Momentary WB			−0.030 (−0.079)	0.083
Long-term WB	**0.640(0.244)**	**< 0.001**		
Individual characteristics and health status				
Age group, years			0.002(0.023)	0.736
Household income, million yen			**−0.080 (−0.140)**	**0.003**
Years of residence			**0.019 (0.202)**	**< 0.001**
Subjective health status			**0.663 (0.186)**	**< 0.001**
High levels of psychological distress			**−0.161 (−0.540)**	**< 0.001**
Perceived environmental characteristics				
Vibrancy	**−0.578 (−0.137)**	**< 0.001**	**0.236 (0.147)**	**< 0.001**
Nature	**0.433 (0.138)**	**< 0.001**		
Relax and clean	**2.305 (0.562)**	**< 0.001**		
Communication	−0.020 (−0.006)	0.868	**0.166 (0.126)**	**0.001**
Location type				
Workplace	−0.693 (−0.026)	0.310		
On the move	**−0.881 (−0.114)**	**0.002**		
Shopping mall/store				
Cafe/restaurant/bar	**0.851 (0.113)**	**< 0.001**		
Cultural/sports/educational facility	**0.449 (0.043)**	**0.049**	**0.240 (0.060)**	**0.025**
Public space	**0.622 (0.113)**	**0.004**		
Other				

*WB* well-being.

Values with a statistical significance level of less than 0.05 are in bold.

## Discussion

Regarding the model fit of SEM, overall, the indices collectively suggest that the model is suitably fitted to the data^11,28^. The study finds a positive association between higher LWB and increased MWB. Urban environment elements such as cafes/restaurants/bars, cultural/sport/education facilities, public spaces, and environments perceived as natural, relaxing, and clean are positively associated with MWB. MWB tends to be lower when on the move and in vibrant environments. LWB is positively associated with cultural/sport/education facilities, vibrancy, and communication in the urban environment. Regarding personal characteristics, people with good health and longer residence exhibit greater LWB levels; however, higher household income and psychological distress were negatively associated with LWB.

### What is the relationship between MWB and LWB?

Our results for research question 1 are in line with prior research^7^. The finding on the relationship between LWB and MWB aligns with those of Weijs-Perrée and colleagues, who discovered that LWB is positively associated with MWB^11^. One perspective is that accumulating moments of MWB could potentially cultivate LWB. On the other hand, the current study demonstrates that overall feelings of well-being may provide a long-term basis for the spontaneous emergence of MWB.

### What are the urban environmental elements associated with MWB and LWB?

Our findings on the relationship between urban environmental factors and LWB and MWB are consistent with prior studies by Weijs-Perrée et al.^11^ and Tan and Lee^29^. Weijs-Perrée et al.. demonstrated a positive correlation between leisure, cafes, bars, and restaurants and individuals’ MWB^11^. Additionally, Tan and Lee identified significant associations among the Malaysian population between life satisfaction and cultural, education-related, and shopping-related places^29^. Mouratidis^30^ revealed that facilities and services, such as cafes and cultural venues, enhance MWB by providing opportunities for leisure activities, social interaction, and physical activity (e.g., walking or cycling), supporting well-being and increasing satisfaction with the living environment. Furthermore, community initiatives, such as workshops and projects to enhance outdoor spaces, contribute to strengthening social bonds among community members^31^. Mouratidis further elaborates that inclusive public and communal areas are essential strategies for augmenting well-being through strategic urban design, as they promote social interaction, physical activity, and community engagement, while fulfilling recreational needs and enhancing overall residential well-being and social cohesion^30^.

This study also highlights the following perceived environmental characteristics in association with positive MWB and LWB. For “nature,” urban parks play a crucial role in promoting well-being, as these greenspaces provide essential restorative ambiance, lower blood pressure, and reduce the risk of depression^32–35^. Moreover, a systematic review revealed that proximity to, and the extent of, waterside spaces in residential areas are correlated with improved mental health and increased physical activity levels^36^.

This study also highlights the following perceived environmental characteristics in association with positive MWB and LWB. For “nature” and “relaxing and clean,” which are associated with MWB, previous studies have reported positive associations between momentary mental well-being and contact with nature, such as seeing trees, hearing birds singing, and seeing the sky^14^. Greenspaces provide essential restorative ambiance, and higher perceived stress has been found to be related to worse mental health for those with lower contact with greenness^37,38^. Moreover, a systematic review revealed that proximity to, and the extent of, waterside spaces in residential areas are correlated with improved mental health and increased physical activity levels^36^. The frequency or duration of exposure to nature may explain the lack of association between nature and LWB. Previous studies have shown that frequent visits to green spaces improve mental health, whereas merely having a connection to nature does not yield these effects^39^. Since our study captures experiences and momentary well-being at a single point in time, continuous exposure to nature might reveal a relationship with LWB.

In terms of “relaxing and clean,” which consists of three characteristics—pleasant design and atmosphere of the space, perceived safety, and clean—a study from the Netherlands demonstrated that satisfaction with the characteristics of a momentary experience (such as air quality, aesthetics, ambiance, scent, ease of access, sufficient parking space, distance to facilities, traffic safety, natural elements, noise, and cleanliness and maintenance of the space) is positively related to MWB^11^. Furthermore, an exploration into the impact of architectural elements on human psychology and physiology indicated that a specific range of color temperature enhances subjective well-being, with lighter colors being perceived as more pleasant, and smooth shapes with rounded edges and graspable size foster positive physiological responses^40^.

Regarding communication, our finding is consistent with previous studies that found that social communication is associated with LWB, such as life satisfaction. Additionally, face-to-face interactions were associated with higher LWB in studies conducted during the COVID-19 pandemic^41–43^. However, in our study, we found no association between communication and MWB. From a momentary perspective, communication is not always positive; there can be instances of arguing, and stressful or negative interactions. In Monninger et al., a positive association between the quality of social interactions and momentary well-being was observed, but no significant effect for the most important interaction partner was found^44^. Since our Communication variable was assessed with two items that capture the relationship with the partner rather than the quality of communication (i.e., “Meeting familiar people” and “Easy to have a conversation”), this may explain why Communication is not significantly associated with MWB.

Furthermore, Kashiwa-no-ha Campus Station area’s urban design guidelines were diligently followed in the area development. These guidelines aim to foster diverse interactions and include measures such as “Open spaces accessible to everyone.” Rather than merely designing spaces as walking spaces, they were conceptualized as networks of diverse plazas where people can linger and engage in activities, thus serving as catalysts for innovation. Such diverse plazas enhance the options of activities to engage in and increase opportunities for encounters, thereby stimulating the possibilities of various interactions^24^.

Contrary to the other positive associations with well-being, being on the move was found to have a negative relationship with MWB, confirming the findings of Weijs-Perrée and colleagues, who identified a negative correlation between relocation and MWB^11^. The relationship between vibrancy and well-being is complex. Our study reveals that while exposure to vibrant environments is positively linked with LWB, it has a negative relationship with MWB. Defining vibrancy as high population density may clarify this observation; it is noted that urbanization is associated with social capital indicators, and population density has a positive association with recent well-being (over the last four weeks), indicating population density as a critical physical attribute of the built environment that affects well-being^45,46^. Conversely, Su found that real-time population levels and noise are inversely related to momentary happiness^47^. Therefore, we consider that higher population density may bring conveniences, such as accessible public transportation and stores; these conveniences can contribute to LWB by enhancing the ease and accessibility of necessary services. However, being in a crowded and/or noisy place might not induce a positive mood in the immediate moment.

### What are the personal characteristics associated with MWB and LWB?

Regarding individual characteristics and health status associated with LWB, longer residence in an area is seen to benefit LWB. Previous studies reported that longer duration of residence positively associates social capital by strengthening attachment to the place and facilitating the formation and maintenance of friendships, which is associated with better mental health outcomes^45,48,49^. On the other hand, our finding of a negative relationship between household income and LWB can be considered in the context of two perspectives: the level of regional economic development and the income range considered. While income significantly impacts well-being in lower-income, developing economies, its effect diminishes in wealthier contexts^50,51^. Our study sample had a high proportion of well-off participants, wherein 56.4% reported household incomes above 8 million yen per year, which is higher than the average annual household income of 5.38 million yen in Kashiwa City. Moreover, research suggests a “satiation point” for income impacting well-being, beyond which increases in income do not significantly enhance well-being. Kahneman found that while well-being increases as annual income reaches the amount of $75,000, it plateaus at higher income levels; however, by 2021, Killingsworth observed a continuous increase in well-being with income, challenging the notion of a universal satiation point^52,53^. Our data, which may reflect a wealthier sample, aligns with the view that in affluent contexts, the relationship between income and well-being may be limited or nuanced.

This study has several limitations that should be considered when evaluating the results. First, the requirement for a smartphone or internet connection introduces participant selection bias. Moreover, we assume that individuals who engaged in this survey and shared their experiences tended to be more conscious of the survey’s themes, which likely resulted in a selection bias. Second, while residential neighborhoods significantly impact the well-being of residents, this study was unable to capture the experiences within indoor home spaces or immediate residential areas. Future studies should explore these contexts to better understand their role in well-being. Third, this study targeted individuals “living in or visiting the Kashiwa-no-ha Campus area,” which may include non-residents, such as commuters. Residents and commuters may interact with the area differently, which could lead to varying perceptions of the environment. While postal code information confirms that 80% of the participants are residents, we were unable to determine whether the remaining participants were commuters or had other purposes for using the area. Future studies should stratify residents and non-residents in their analysis. Fourth, this study is restricted to a single urban area, limiting the ability to generalize findings to other contexts with different cultural, environmental, or design conditions. Future research is needed to validate these findings across different regions. Fifth, the cross-sectional design of the study leaves causality indeterminate. Sixth, personality and other perceived factors, such as perceived thermal comfort, perceived soundscapes, including noise or unpleasant soundscapes, were not considered in this study, presenting a gap that future studies can address. Seventh, our dataset contains two levels of hierarchy: individual and experience levels. As such, using the Multilevel Structural Equation Model (MSEM) seemed to be the appropriate choice; however, in MSEM, aggregate experience level variables need to be applied to individual level variables. Since aggregating at the item level does not align with our research questions, we decided to conduct our analysis using a simple SEM instead of a multilevel approach.

## Conclusion

This paper utilizes the ESM method to offer insights into the relationship between urban environments and both long-term and momentary well-being. It is evident that MWB is crucial and is associated with the urban environment, while LWB also holds significant importance. Our findings reveal that public spaces, cafes, cultural facilities, and so on, significantly contribute to well-being, highlighting their importance in urban planning as third places—such as cafes, parks, and community facilities—that promote a positive mood and happiness. On the other hand, experiences of being “on the move” are associated with lower MWB, indicating the need for thoughtfully designed transportation and road environments. These designs should not only facilitate ease of movement but also enhance the overall urban experience. Moreover, our study suggests that creating environments that encourage easy communication, relaxation, and a connection to nature can significantly improve well-being. These elements serve as critical interventions in urban planning, helping create spaces that not only meet the physical needs of residents but also support their emotional and psychological well-being. Urbanization challenges future development by potentially altering how people interact with their environment. Integrating these insights into planning helps prioritize well-being.

## Declaration of generative AI and AI-assisted technologies in the writing process

During the preparation of this work, the authors used ChatGPT for grammar checking. After using this tool, the authors reviewed and edited the content as needed and take(s) full responsibility for the content of the publication.

## Electronic supplementary material

Below is the link to the electronic supplementary material.

Supplementary Material 1

## Data Availability

The data that support the findings of this study are confidential and not publicly available due to privacy and ethical restrictions. For further information regarding the data, please contact yuruchen9411@gmail.com.
