# Supplementary material for: Perceived urban environment elements associated with momentary and long-term well-being: An experience sampling method approach
Source: Sci Rep. 2025 Feb 5;15:4422. doi: 10.1038/s41598-025-88349-x (PMC11799535; doi:10.1038/s41598-025-88349-x)
Supplement: Supplementary file 1 — Supplementary Material 1 [file 41598_2025_88349_MOESM1_ESM.docx]

**Supplementary Materials**

[**1.** **Study area (Kashiwa-no-ha area)** 1](#_Toc185508278)

[**2.** **Procedural diagram of the study** 2](#_Toc185508279)

[**3.** **Online questionnaire** 3](#_Toc185508280)

[**4.** **Experience Sampling Method survey** 7](#_Toc185508281)

1. **Study area (Kashiwa-no-ha area)**

The Kashiwa-no-ha area refers to the surrounding area of Kashiwa-no-ha Campus Station. Kashiwa-no-ha Campus Station was established in 2005 with the opening of the Tsukuba Express line. Since then, the area around the station has been developed with public squares, commercial facilities, residential buildings etc. As there is no specific definition (in terms of range or distance) for the "Kashiwa-no-ha area," it usually refers to the region within a 2 km radius from the station (Figures 1 and 2).


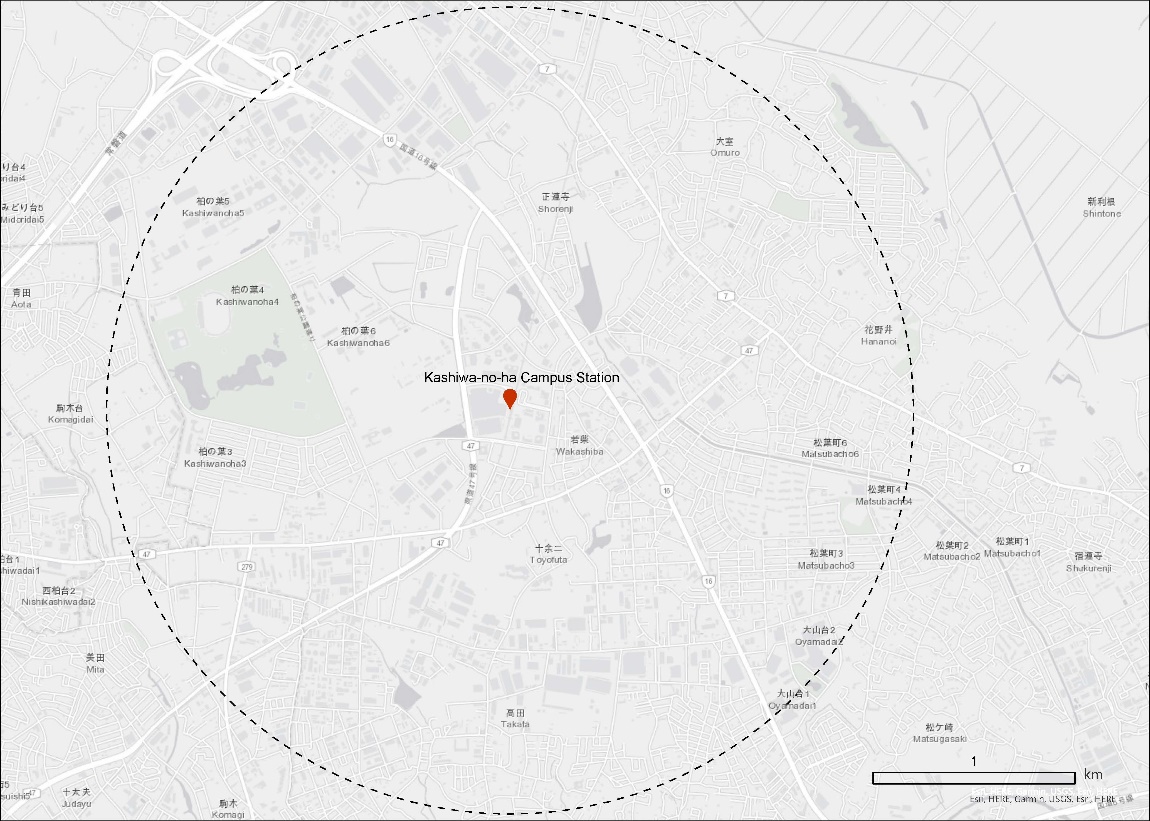


Figure 1. Kashiwa-no-ha Campus Station area

Kashiwa-no-ha Campus Station


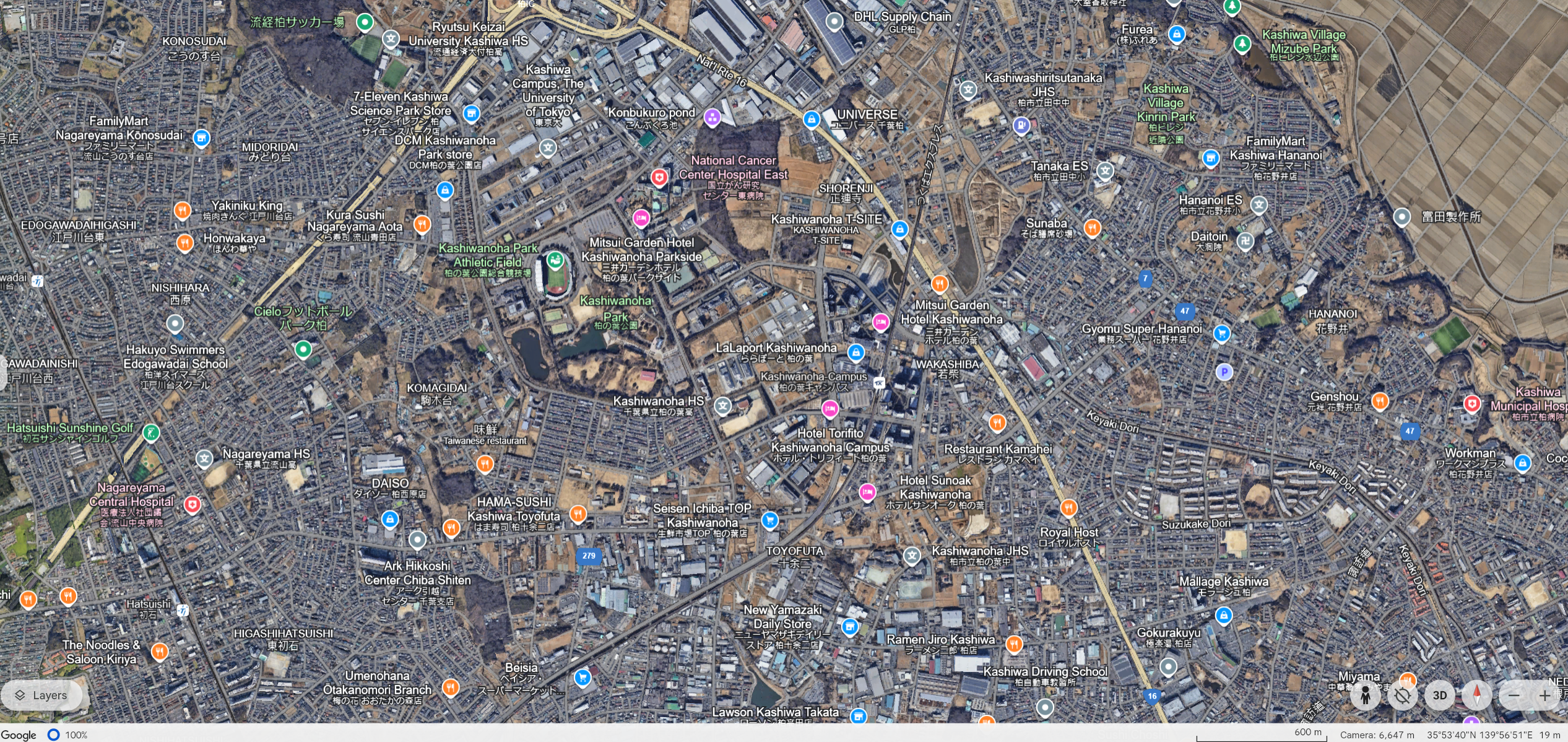


Figure 2. Kashiwa-no-ha Campus Station area with labeled locations

Google Earth. (2024). Kashiwa, Chiba, Japan (Version 10.69.0.1). Latitude 35°53'40"N, Longitude 139°56'51"E. Retrieved December 18, 2024, from <https://www.google.com/earth>

1. **Procedural diagram of the study**

**
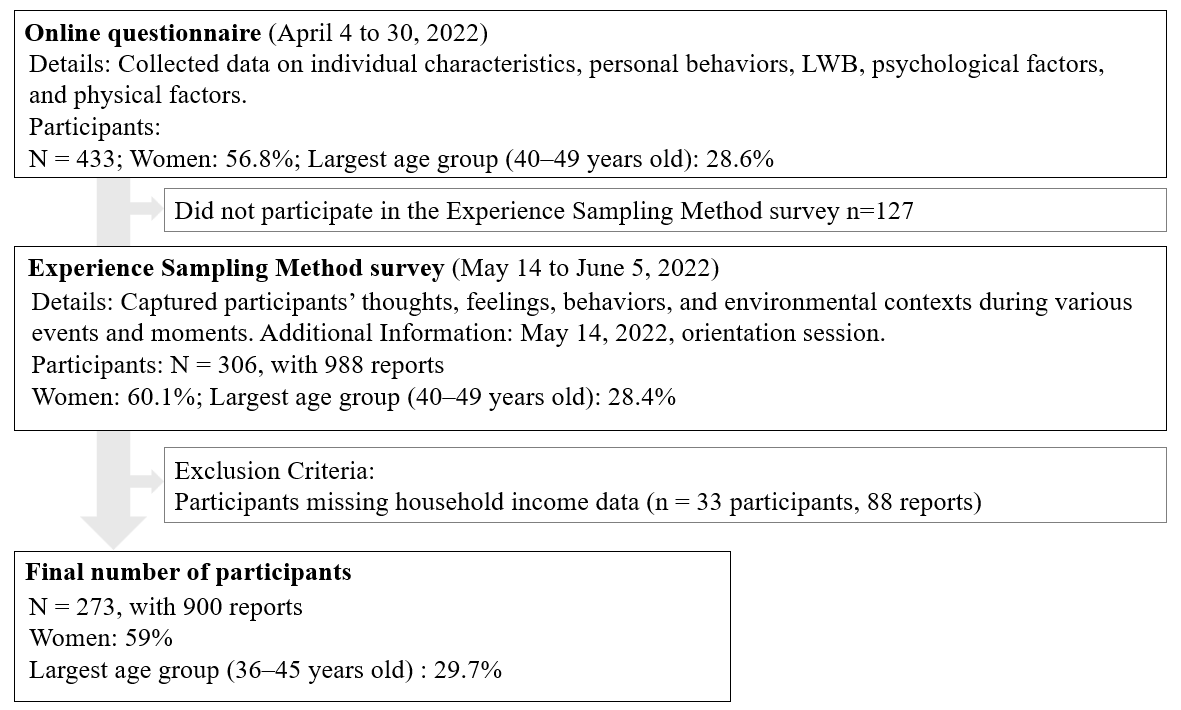
**

Figure 3. Procedural diagram of the study

1. **Online questionnaire**

**Satisfaction and Well-being**

For the following questions, please select the option that best describes your situation on a scale of 0 to 10, where 10 is the best state and 0 is the worst.

**How satisfied are you with your life overall?**
*Select one of the following:*

0 (Not satisfied at all) to 10 (Very satisfied)

**To what extent do you feel that the things you do in life are valuable?**
*Select one of the following:*

0 (Not valuable at all) to 10 (Very valuable)

**To what degree do you feel you are currently happy? (Score "0" for "Very unhappy" and "10" for "Very**

**happy.")?**
*Select one of the following:*

0 (Very unhappy) to 10 (Very happy)

**I understand the purpose of my life.**
*Select one of the following:*

0 (Not at all) to 10 (Very much)

**Community and Neighborhood**

**What is your postal code?**
*Enter the seven-digit postal code.*
(Example: Enter "2730005" for 273-0005. If unknown, enter "0000000".)

**How long have you lived in your current residence?**
*Enter the number of years:*
(If less than one year, enter "0".)

**Demographic Information**

**Gender:**
*Select one of the following:*

Men

Women

Prefer not to answer

**Age:**
*Enter your age in years.*

**Height:**
*Enter your height in cm.*

**Weight:**
*Enter your weight in kg.*

**What is the highest level of education you have completed?**
*Select one of the following:*

Elementary/junior high school

High school

College/technical school

University

Graduate school

Don’t know

Prefer not to answer

**What is your employment status?**
*Select one of the following:*

Employed (full-time)

Employed (part-time, casual)

Unemployed (previously employed)

Never worked

**What is your marital status?**
*Select one of the following:*

Married (includes partner)

Single

Divorced

Widowed

**What type of housing do you live in?**
*Select one of the following:*

Owned house (detached)

Owned apartment (condominium)

Public rental housing

Private rental housing (detached)

Private rental housing (apartment)

Rented room/house

**How many people live with you, including yourself?**
*Select one of the following:*

1 (living alone)

2

3

4

5

6

7

8

9

10 or more

**Please select the number of household members who live with you and share your household expenses (including yourself).**

Please select only one of the following:

1 person (yourself)

2 people

3 people

4 people

5 people

6 people

7 people

8 people

9 people

10 or more people

**Household Income (before taxes)**

This refers to the total income earned by your entire household in one year, including both earned income and other types of income (such as allowances from parents, property income like rent, child allowances, etc.).

Please select only one of the following:

Less than 2,000,000 yen

2,000,000 yen to less than 4,000,000 yen

4,000,000 yen to less than 6,000,000 yen

6,000,000 yen to less than 8,000,000 yen

8,000,000 yen to less than 10,000,000 yen

10,000,000 yen to less than 15,000,000 yen

More than 15,000,000 yen

Prefer not to answer

Don't know

**How is your current health condition?**

Please select only one of the following:

Very good

Fairly good

Not very good

Poor

**In the past 30 days, how often have you experienced the following?**

Please select the most appropriate response for each item (options: Never, Only a little, Sometimes, Often, Always):

Have you felt nervous?

Have you felt hopeless?

Have you felt restless or unable to relax?

Have you felt so down that nothing could cheer you up?

Have you felt like everything you do takes great effort?

Have you felt like you are a worthless person?

# **Experience Sampling Method survey**

Please answer the questions about places, experiences, and impressions where you felt comfortable or uncomfortable.
(Estimated time to complete: about 5 minutes)

**Was this experience at home?**

- Yes
- No

**Please move the red pin on the map to as accurately as possible indicate where you felt comfortable or uncomfortable.**
(*You can zoom in or out on the map*)
Instructions for using the map:

- Place two fingers on the screen and move them in any direction
- To zoom in, spread your two fingers apart on the screen
- To zoom out, bring your two fingers closer together

**Were you able to place the red pin on the map without any problems?**
*Please answer only if the response to 'Was this experience at home?' was 'No':*

- Yes
- No

**Was the place indoors or outdoors?**
*Please select only one of the following:*

- Indoors
- Outdoors

**How were you feeling at the time of the experience?**
At that moment, I felt…
(*Please rate on a scale from 0 to 6, where 6 is the most ideal state for you and 0 is the worst*)

- Well/Unwell
- Contentment/Discontentment
- Relaxed/Tense
- Calm/Agitated
- Tired/Awake
- Without energy/Full of energy

**What type of place was this?**
*Please answer only if the experience occurred indoors:*

- Shopping mall/store/supermarket
- Restaurant
- Café/bar
- Public (Cultural/sports/educational) facility (e.g., museum, library, community center)
- Sports facility
- Inside a station
- Event venue
- Workplace

**Regarding the place, please select the most appropriate response:**
(*Please answer only if the experience occurred indoors*)
For each statement, choose the option that applies:

- Lively and crowded
- Few people, calm atmosphere
- Meeting familiar people
- Pleasant design and atmosphere of the space
- Easy to have a conversation
- Feeling safe
- Clean
- Easy to access
- Greenery such as trees and plants
- Waterfront such as a river or the ocean
- Pleasant natural light (e.g. sunlight filtering through trees)
- Pleasant artificial light (e.g. color and arrangement of lighting)
- Benches and chairs to sit or rest
- Signs providing directions/information about the location
- Pleasant sounds and music
- Parking lots and bicycle parking areas

**What type of place was this?**
*Please answer only if the experience occurred outdoors:*

- Plaza
- Park
- Waterfront area
- Event venue
- Restaurant
- Café
- Sports facility
- Public (Cultural/sports/educational) facility (e.g., museum, library, community center)
- Inside a station
- On the move
- Workplace

**Regarding the place, please select the most appropriate response:**
(*Please answer only if the experience occurred outdoors*)
For each statement, choose the option that applies:

- Lively and crowded
- Easy to walk, makes you want to walk
- Meeting familiar people
- Pleasant design/atmosphere of surrounding buildings
- Pleasant design and atmosphere of the space
- Easy to have a conversation
- Feeling safe
- Clean
- Easy to access
- Greenery such as trees and plants
- Waterfront such as a river or the ocean
- Pleasant natural light (e.g. sunlight filtering through trees)
- Pleasant artificial light (e.g. color and arrangement of lighting)
- Benches and chairs to sit or rest
- Signs providing directions/information about the location
- Pleasant sounds and music
- Parking lots and bicycle parking areas

Note. The environmental characteristics “Few people, calm atmosphere” for indoors experience and “Easy to walk, makes you want to walk,” “Pleasant design/atmosphere of surrounding buildings” for outdoors experience were not included in this analysis because they were not shared survey items across both indoor and outdoor contexts.
